# Select Dietary Phytochemicals Function as Inhibitors of COX-1 but Not COX-2

**DOI:** 10.1371/journal.pone.0076452

**Published:** 2013-10-03

**Authors:** Haitao Li, Feng Zhu, Yanwen Sun, Bing Li, Naomi Oi, Hanyong Chen, Ronald A. Lubet, Ann M. Bode, Zigang Dong

**Affiliations:** 1 The Hormel Institute, University of Minnesota, Austin, Minnesota, United States of America; 2 The National Institutes of Health, National Cancer Institute, Bethesda, Maryland, United States of America; University of Padua, Italy

## Abstract

Recent clinical trials raised concerns regarding the cardiovascular toxicity of selective cyclooxygenase-2 (COX-2) inhibitors. Many active dietary factors are reported to suppress carcinogenesis by targeting COX-2. A major question was accordingly raised: why has the lifelong use of phytochemicals that likely inhibit COX-2 presumably not been associated with adverse cardiovascular side effects. To answer this question, we selected a library of dietary-derived phytochemicals and evaluated their potential cardiovascular toxicity in human umbilical vein endothelial cells. Our data indicated that the possibility of cardiovascular toxicity of these dietary phytochemicals was low. Further mechanistic studies revealed that the actions of these phytochemicals were similar to aspirin in that they mainly inhibited COX-1 rather than COX-2, especially at low doses*.*

## Introduction

Consistent clinical studies have indicated that long-term administration of COX-2 inhibitors is associated with an enhanced risk of experiencing adverse cardiovascular events [1,2]. Although the exact mechanism still remains unclear, accumulating evidence supports the idea that COX-2 plays a cardioprotective role after cardiac injury [3-5]. Functional recovery after induced cardiac injury was improved in COX-2 transgenic mouse, but was greatly reduced by deficiency of COX-2. In the search to identify promising cancer chemopreventive agents, dietary phytochemicals have emerged as potential agents based on their observed anticancer activities as well as perceived safety [6]. Some mechanistic studies revealed that active dietary factors, such as EGCG, curcumin or resveratrol, might suppress carcinogenesis by targeting COX-2 [7-13]. One question that has been raised is why the lifelong use of phytochemicals that likely inhibit COX-2 has reportedly not been associated with adverse cardiovascular side effects. We hypothesize that those naturally occurring compounds might share a similar mechanism of action with aspirin, and might preferentially target COX-1 rather than COX-2. To test this idea, we selected a library of well-known active dietary factors and evaluated their potential cardiovascular toxicity as well as their effects on COX activity*.*

## Materials and Methods

### Reagents and chemicals

The COX-1 (#4841) and COX-2 (#12282) antibodies were purchased from Cell Signaling Technology (Beverley, MA). The antibody against β-actin (SC47778) was from Santa Cruz Biotechnology (Santa Cruz, CA). Interleukin-1 beta (IL-1β) was from Millipore (Billerica, MA). All other chemicals were obtained from Sigma-Aldrich (St. Louis, MO) unless otherwise specified.

### Cell culture

COX-2 Wild-type (COX-2^+/+^) and COX-2 knockout (COX-2^-/-^) mouse embryonic fibroblasts (MEFs) were kind gifts from Drs. Jeff Reese and Sudhansu K. Dey (University of Kansas Medical Center) [14]. The cells were derived from COX-2 knockout mice supplied by Drs. Joseph E. Dinchuk and James M. Trzaskos (DuPont Merck Pharmaceutical Co.) [15]. The cells were cultured in monolayers at 37 °C, 5% CO_2_ using Dulbecco’s modified Eagle’s medium containing 10% FBS, 1% penicillin/streptomycin and 2mM L-glutamine. All other cell lines used in this study were obtained from the American Type Culture Collection (Manassas, VA, USA) and maintained following their instructions. Cells were cytogenetically tested and authenticated before the cells were frozen. The passage number was routinely limited to approximately 20 and morphology monitored with each passage.

### Cell viability assay

Cells were seeded in 96-well-plate at a density of 5000 cells per well and allowed to incubate at 37 °C for 24 h for attachment. After drug treatment for 8 h, 20 µL CellTiter96 Aqueous One Solution (Invitrogen, Carlsbad, CA) were added, and cells were further incubated for 1 h at 37 °C. Finally, the optical density was determined at 492 nm.

### Measurement of NO

HUVEC cells (6×10^5^) were seeded in a six-well plate in the presence of 10% FBS. At 70-80% confluence, cells were pretreated with vehicle or individual compounds in 1 mL fresh medium for 2 h. After that, cells were incubated or not with IL-1β (17.5 ng/mL) for another 8 h. Supernatant fractions were collected for measurement of nitric oxide (NO). NO concentration was measured as nitrite using the Nitrate/Nitrite colorimetric assay kit (Cayman Chemicals, Ann Arbor, MI).

### In vitro COX enzyme assay

COX activity was evaluated using a COX Inhibitor Screening Kit from Cayman Chemical Company (Ann Arbor, MI) according to the manufacturer’s instructions.

### Thromboxane B_2_ (TXB_2_) and 6-keto prostaglandin F_1α_ (6-keto PGF_1α_) assay

Cells (6×10^5^) were seeded in a six-well plate in the presence of 10% FBS. At 70-80% confluence, cells were pretreated with vehicle or individual compounds in 1 mL fresh medium for 2 h. After that, cells were or were not incubated with IL-1β (17.5 ng/mL) for another 8 h. Supernatant fractions were collected for prostaglandin measurement using enzyme immunoassay kits (Cayman Chemical Company).

### Western blot analysis

Protein samples (20 µg) were resolved by SDS-PAGE and transferred to Hybond C nitrocellulose membranes (Amersham Corporation, Arlington Heights, IL). After blocking, the membranes were probed with primary antibodies (1:1000) overnight at 4 °C. The targeted protein bands were visualized using an enhanced chemiluminescence reagent (Amersham Corporation) after hybridization with a secondary antibody conjugated with horseradish peroxidase.

### Statistical analysis

All experiments were performed at least three times independently. Statistical analysis was performed using the Prism statistical package. Turkey’s t-test was used to compare data between two groups. One-way ANOVA and the Bonferroni correction were used to compare data between three or more groups. Values are expressed as means ± S.E.M. and a *p* < 0.05 was considered statistically significant.

## Results

### Evaluation of potential cardiovascular toxicity

**Figure 1:**
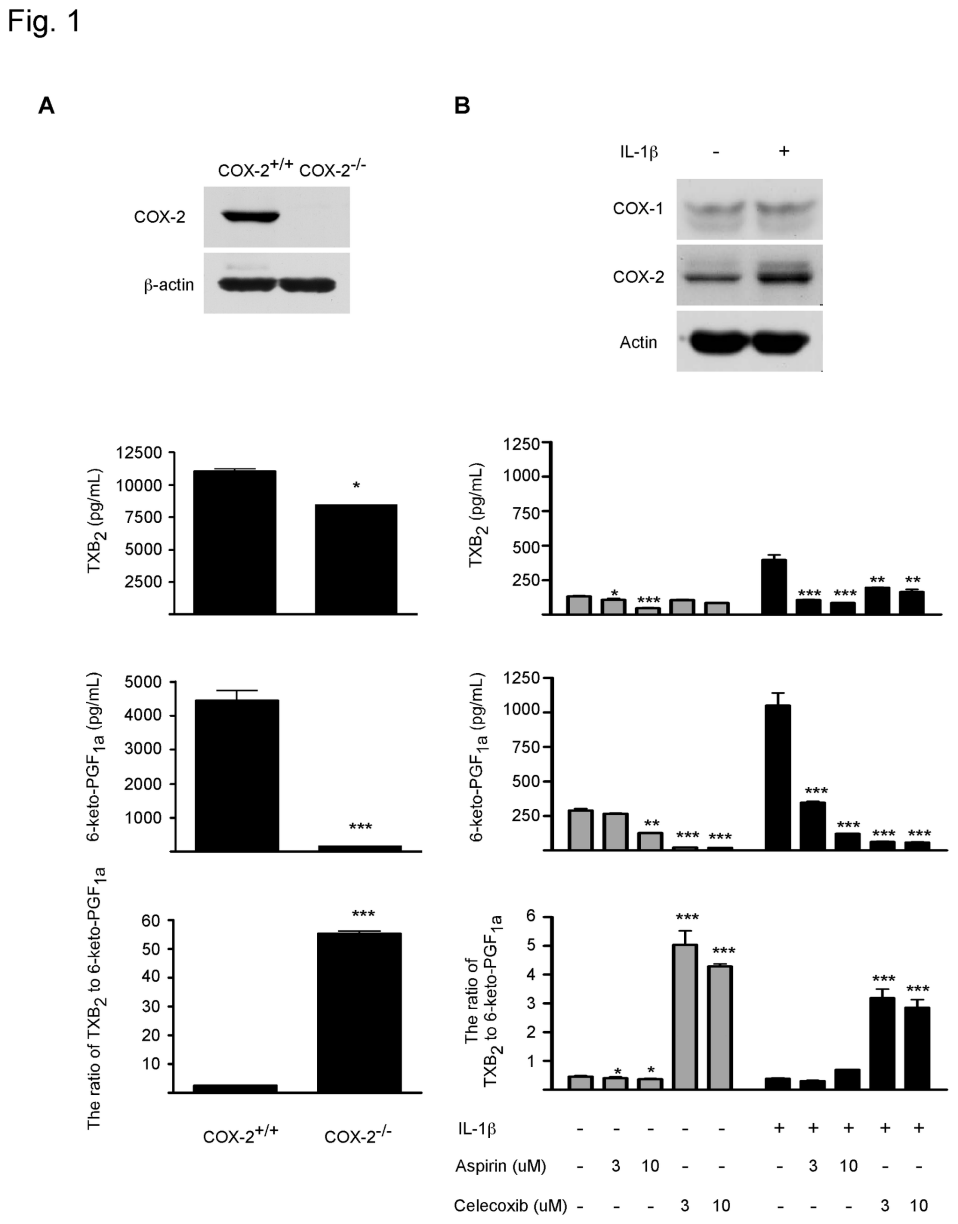
Effect of COX-2 inactivation on TXB_2_ and 6-keto-PGF_1α_ in murine embryo fibroblasts. A. COX-2 deficiency enhanced the ratio of thromboxane B_2_ to 6-keto prostaglandin F_1α_. Western blot analysis of murine embryo fibroblasts (MEFs). Cells (6×10^5^) were seeded in a six-well plate in the presence of 10% FBS. When cell reached 70-80% confluence, fresh culture medium (1 mL/well) was added. After further incubation for 24 h, supernatant fractions were collected for prostaglandin measurement. Data are presented as means ± S.E.M (n = 4) and the asterisk(s) indicate a significant (*, *p* < 0.05; ***, *p* < 0.001) difference versus the COX-2 wildtype group. B. COX-2 inhibition enhanced the ratio of thromboxane B_2_ to 6-keto prostaglandin F_1α_. Human umbilical vein endothelial cells (HUVECs) were seeded in a six-well plate in the presence of 10% FBS. At 70-80% confluence, cells were pretreated with 1 mL fresh medium containing DMSO or each individual compound for 2 h, and then IL-1β (17.5 ng/mL) was added together with each individual compound for another 8 h incubation. Supernatant fractions were collected for prostaglandin measurement. Data are presented as means ± S.E.M. (n = 4) and the asterisk(s) indicate a significant (*, *p* < 0.05; **, *p* < 0.01; ***, *p* < 0.001) difference versus Control.

The imbalance between COX-1-derived pro-thrombotic thromboxane A_2_ (TXA_2_) and COX-2-relateded anti-thrombotic prostacyclin (PGI_2_) production has long been suspected to contribute to cardiovascular side effects of COX-2 inhibitors [16,17]. COX-2^-/-^ mice are more prone to cardiovascular risk than wild type mice, evidenced by increased cardiac ischemia and/or reperfusion injury [18]. Therfore, we firstly examined this idea in wildtype (COX2^+/+^) or knockout (COX^-/-^) mouse embryonic fibroblasts (MEFs, Figure 1A). COX-2 deficiency enhanced the ratio of thromboxane B_2_ (TXB_2_, the stable breakdown product of TXA_2_) to 6-keto prostaglandin F_1α_ (6-keto PGF_1α_, the hydrolysis product of PGI_2_) by 22-fold. We further tested such hypothesis in an *in vitro* model using human umbilical vein endothelial cells (HUVECs). Again, the ratio of TXB_2_/6-keto-PGF1_α_ was dramatically increased by the selective COX-2 inhibitor celecoxib, but not by aspirin, which is known to target COX-1 rather than COX-2 (Figure 1B). All of these findings support the reliability of the ratio of thromboxane B_2_ to 6-keto prostaglandin F_1α_ as a biomarker for COX-2 inhibition-related cardiovascular toxicity.

**Figure 2:**
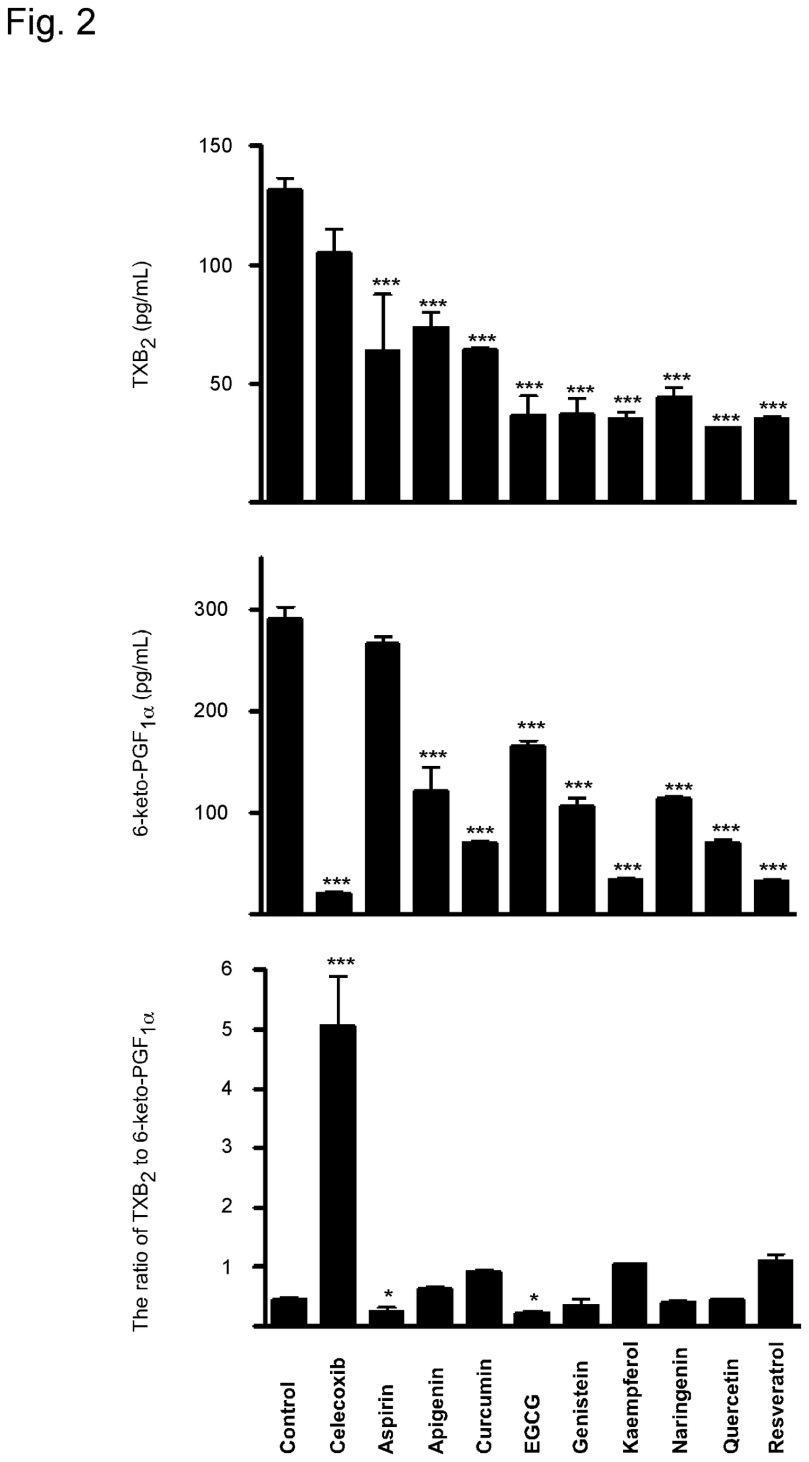
Effects of dietary phytochemicals on (A) TXB_2_ and (B) 6-keto-PGF_1_α under physiological conditions. HUVECs were seeded in a six-well-plate (6×10^5^ cells per well). At 70-80% confluence, 1 mL fresh medium containing DMSO or 3 µM of each individual compound was added and cells were further incubated for 8 h. Supernatant fractions were collected for prostaglandin measurement. (C) The ratio of TXB_2_/6-keto-PGF_1_α. Data are presented as means ± S.E.M (n = 4) and the asterisk(s) indicate a significant (*, *p* < 0.05; ***, *p* < 0.001) difference versus the vehicle control group.

The potential cardiovascular toxicity of dietary phytochemicals was then evaluated in this *in vitro* model. Considering their clinically achievable serum concentrations, all dietary factors were administrated at 3 µM [19-27]. Compared with celecoxib, all of dietary factors only weakly disturbed the ratio of TXB_2_/6-keto-PGF1_α_ (Figure 2).

**Figure 3:**
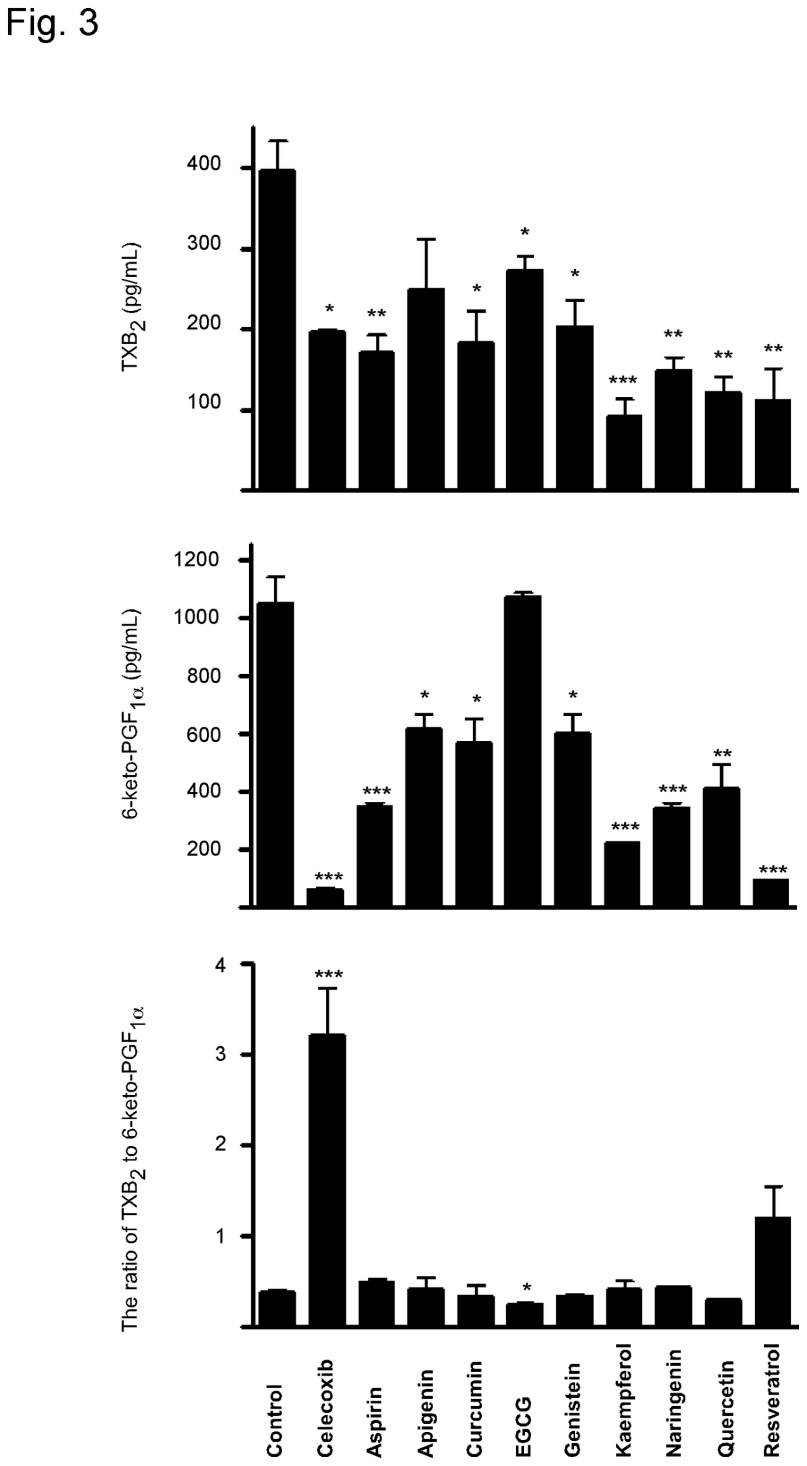
Effects of dietary phytochemicals on (A) TXB_2_ and (B) 6-keto-PGF_1_α under pro-inflammatory conditions. HUVECs were seeded in a six-well-plate (6×10^5^ cells per well). At 70-80% confluence, cells were pretreated with 1 mL fresh medium containing DMSO or 3 µM of each individual compound for 2 h and then IL-1β (17.5 ng/mL) was added together with each individual compound for another 8 h incubation. Supernatant fractions were collected for prostaglandin measurement. (C) The ratio of TXB_2_/6-keto-PGF_1_α. Data are presented as means ± S.E.M. (n = 4) and the asterisk(s) indicate a significant (*, *p* < 0.05; **, *p* < 0.01; ***, *p* < 0.001) difference versus Group 1 (IL-1β).

Considering the fact that people usually take selective COX-2 inhibitors to relieve pain and reduce inflammation, HUVECs were treated with IL-1β, an inflammatory cytokine implicated in vascular diseases, to mimic pro-inflammatory conditions. Stimulation of cells with IL-1β resulted in a remarkable increase in COX-2 expression as well as 6-keto-PGF1_α_ synthesis. Similar to physiological conditions, the ratio of TXB_2_/6-keto-PGF1_α_ was significantly enhanced by celecoxib, whereas was only weakly affected by aspirin as well as by dietary factors (Figure 3).

**Figure 4:**
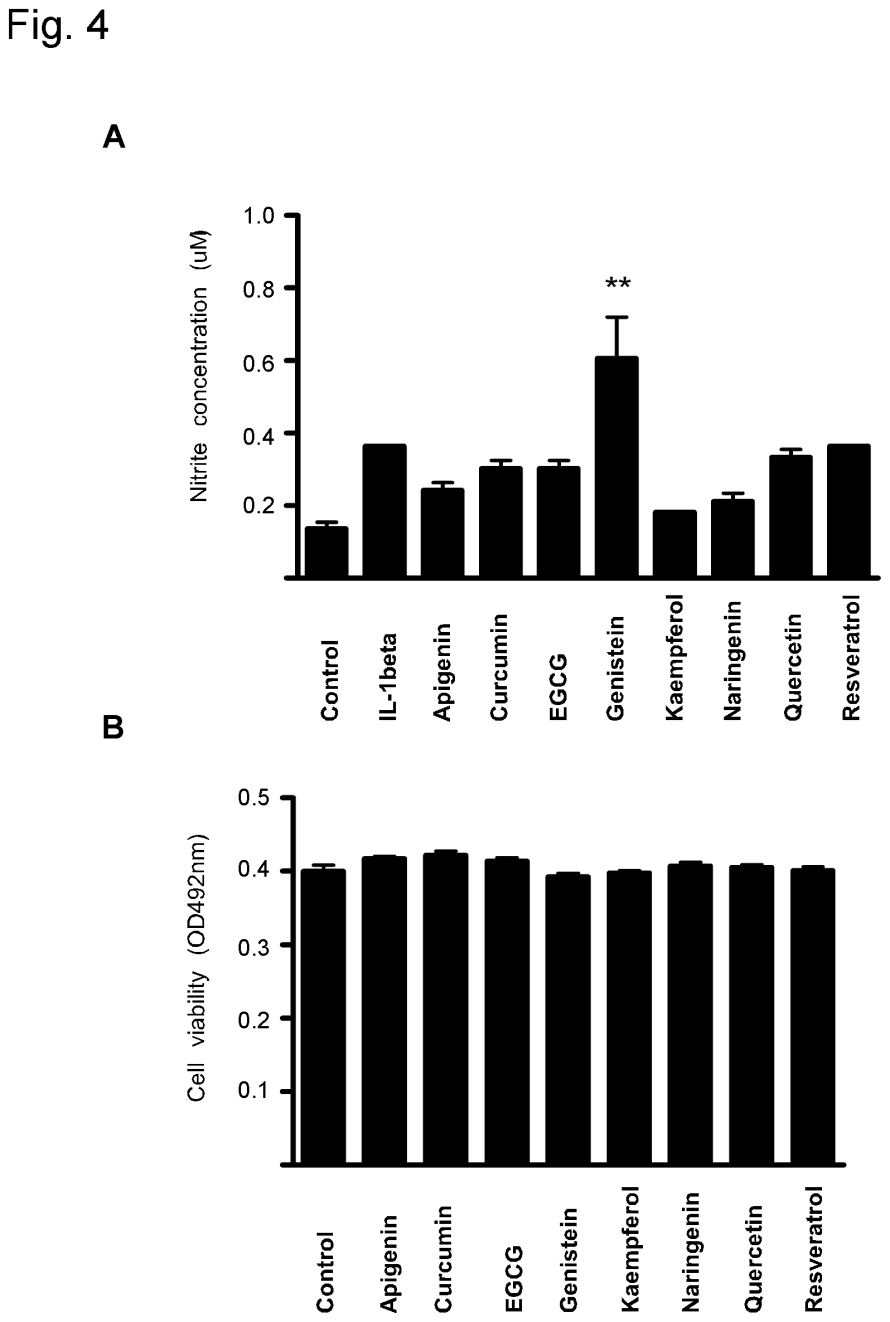
Effects of dietary phytochemicals on (A) nitric oxide (NO) production and (B) cell viability. NO concentration was measured as nitrite using the Nitrate/Nitrite colorimetric assay kit as described in “Materials and Methods”. Data are presented as means ± S.E.M. (n = 3) and the asterisk(s) indicate a significant (**, *p* < 0.01) difference versus IL-1β group. Cell viability was tested as described in “Materials and Methods”. Data are presented as means ± S.E.M. (n = 3) and the asterisk(s) indicate a significant (*, *p* < 0.05; **, *p* < 0.01; ***, *p* < 0.001) difference versus control (DMSO).

In addition to COX-2-related PGI_2_, endothelial-derived nitric oxide (NO) also acted as an endogenous vasodilator and protected the blood vessel wall by inhibiting platelet aggregation. In this study, we observed that IL-1β treatment caused a 2.6 fold increase in NO production compared with the control group. More importantly, most dietary phytochemicals had no effect on NO release (Figure 4A). We also excluded generalized cytotoxicity by examining the effects of dietary phytochemicals on cell viability and found that they had little effect on HUVEC cell viability after 8 hours of treatment (Figure 4B). Taken together, these findings suggested that the possible cardiovascular toxicity of dietary phytochemicals is low.

### Determination of the effect of phytochemicals on COX activity in vitro

The differential effects between dietary factors and celecoxib on cardiovascular toxicity biomarkers indicated that these dietary phytochemicals might not be specific COX-2 inhibitors. Accordingly, we determined their effect on COXs activity using a COX inhibitor screening assay kit. Results clearly indicated that most of the compounds are COX-1 inhibitors with a mild to moderate COX-1 selectivity index (Table 1). Among eight dietary phytochemicals, six selectively inhibited COX-1 activity rather than COX-2. Another two flavonols (naringenin and quercetin) are likely not COX1/2 inhibitors because their 50% inhibitory concentrations (IC_50_) against COX1/2 activity was higher than 400 µM.

**Table 1:** Inhibition of COX activity by dietary phytochemicals.

**Compound**	**Natural Source**	**COX-1 IC_50_ (µM)**	**COX-2 IC_50_ (µM)**
Celecoxib		95.4±12.7	0.02±0.009
Aspirin	White willow	4.7±1.2	18.1±4.3
Apigenin	Celery	94.1±12.3	146.4±16.5
Curcumin	Curry	330.1±34.3	NA
Genistein	Soybean	9.9±2.3	256.2±35.7
EGCG	Green tea	17.9±4.2	28.6±3.8
Kaempferol	Broccoli	110.6±7.5	235.8±19.7
Naringenin	Orange	NA	NA
Quercetin	Black tea	NA	NA
Resveratrol	Grape	3.4±1.1	8.5±2.3

The effect of selected dietary factors on COX activity was evaluated using a COX Inhibitor Screening Kit (Cayman Chemical) according to the manufacturer’s instructions. IC_50_ values were calculated from a plot of percent inhibition versus the logarithm of concentration. Data are presented as means ± S.E.M. of 3 independent experiments.

## Discussion

In this study, the cardiovascular safety of selected dietary factors was systemically evaluated for the first time. Our data indicated that the possible cardiovascular toxicity of dietary phytochemicals was low because the compounds tested might share a mechanism of action similar to aspirin and most appeared to preferentially target COX-1 rather than COX-2.

During the course of this study, EGCG, an active ingredient in green tea, exhibited an unexpected cardioprotective property and might merit further investigation. Among dietary factors studied, EGCG exhibited the most potent inhibitory effect against the ratio of TXB_2_/6-keto-PGF1_α_ either under physiological or pathological conditions. This finding was consistent with several recent epidemiologic studies, which suggested regular consumption of green tea might provide cardioprotective effects [28,29]. This unanticipated finding provides critical insight into the potential application of green tea for cardioprotection.

Aspirin at low dose (81 mg per day) is widely accepted to be able to provide both cardioprotective and chemopreventive effects [17,23,30]. However, pharmacokinetic data analysis revealed that at this dose, aspirin might mainly targets COX-1 rather than COX-2, because the maximal serum concentration achieved was well below the reported whole blood COX-2 IC_50_ values [31,32]. In this study, we confirmed that most natural product-based compounds were COX-1, rather than COX-2 selective inhibitors. This raised the question of whether those natural occurring compounds exert their chemopreventive activity, at least in part, by targeting COX-1. Although no conclusion can be drawn due to insufficient data at this time, accumulating evidence suggests that COX-1 is involved in carcinogenesis [33-36]. For example, overexpression of COX-1 leads to tumorigenic transformation, whereas genetic disruption of *ptgs-1* greatly reduced cancer incidence both in skin and colon. Although COX-1 is now becoming a target to be reconsidered for cancer prevention or treatment, selective COX-1 inhibition is still a controversial issue. For example, inhibition COX-1 has been strongly implicated in the gastric ulceration and bleeding induced by non-steroidal anti-inflammatory drugs (NSAIDs) because people believe that COX-1 is responsible for the prostaglandins essential for normal mucosal physiology in gut. As no gastrointestinal toxicity data were collected in this study, whether these phytochemicals cause gastrointestinal bleeding is still unknown and further study in these areas is required.
